# COVID-19-related anosmia is associated with viral persistence and inflammation in human olfactory epithelium and brain infection in hamsters

**DOI:** 10.1126/scitranslmed.abf8396

**Published:** 2021-05-03

**Authors:** Guilherme Dias de Melo, Françoise Lazarini, Sylvain Levallois, Charlotte Hautefort, Vincent Michel, Florence Larrous, Benjamin Verillaud, Caroline Aparicio, Sebastien Wagner, Gilles Gheusi, Lauriane Kergoat, Etienne Kornobis, Flora Donati, Thomas Cokelaer, Rémi Hervochon, Yoann Madec, Emmanuel Roze, Dominique Salmon, Hervé Bourhy, Marc Lecuit, Pierre-Marie Lledo

**Affiliations:** 1 Lyssavirus Epidemiology and Neuropathology Unit, Institut Pasteur, 75015 Paris, France.; 2 Perception and Memory Unit, Institut Pasteur, CNRS UMR3571, 75015 Paris, France.; 3 Biology of Infection Unit, Institut Pasteur, Paris, France; Inserm U1117, 75015 Paris, France.; 4 Otolaryngology-head and Neck Surgery Department, Hopital Lariboisiere, Assistance Publique - Hôpitaux de Paris, Inserm U1141, Université de Paris, 75010 Paris, France.; 5 Institut de l'Audition, Institut Pasteur, Paris, France; Inserm U1120, 75012 Paris, France.; 6 Emergency Department, Hôpital Lariboisière, Assistance Publique - Hôpitaux de Paris, Université de Paris, 75010 Paris, France.; 7 Laboratory of Experimental and Comparative Ethology, Université Sorbonne Paris Nord, Villetaneuse, France.; 8 Plateforme Technologique Biomics – Centre de Ressources et Recherches Technologiques (C2RT), Institut Pasteur, 75015 Paris, France.; 9 Hub de Bioinformatique et Biostatistique – Département Biologie Computationnelle, Institut Pasteur, USR 3756 CNRS, 75015 Paris, France.; 10 National Reference Center for Respiratory Viruses, Institut Pasteur, 75015 Paris, France.; 11 Molecular Genetics of RNA Viruses, CNRS UMR3569, University of Paris, Institut Pasteur, 75015 Paris, France.; 12 Otolaryngology-head and Neck Surgery Department, GHU Pitié-Salpêtrière, Assistance Publique-Hôpitaux de Paris, Sorbonne Université, 75013 Paris, France.; 13 Emerging Diseases Epidemiology Unit, Institut Pasteur, 75015 Paris, France.; 14 Sorbonne Université, AP-HP, Hôpital de la Pitié-Salpêtrière, Département de Neurologie, Inserm U1127, CNRS UMR 7225, Institut du Cerveau, 75013 Paris, France.; 15 Infectious Diseases and Immunology Department, Cochin Hotel Dieu Hospital, Assistance Publique - Hôpitaux de Paris, Université de Paris, 75015 Paris, France.; 16 Université de Paris, Necker-Enfants Malades University Hospital, Division of Infectious Diseases and Tropical Medicine, Institut Imagine, AP-HP, 75015 Paris, France.

## Abstract

Whereas recent investigations have revealed viral, inflammatory and vascular factors involved in SARS-CoV-2 lung pathogenesis, the pathophysiology of neurological disorders in COVID-19 remains poorly understood. Olfactory and taste dysfunction are common in COVID-19, especially in mildly symptomatic patients. Here, we conducted a virologic, molecular, and cellular study of the olfactory neuroepithelium of seven patients with COVID-19 presenting with acute loss of smell. We report evidence that the olfactory neuroepithelium may be a major site of SARS-CoV2 infection with multiple cell types, including olfactory sensory neurons, support cells, and immune cells, becoming infected. SARS-CoV-2 replication in the olfactory neuroepithelium was associated with local inflammation. Furthermore, we showed that SARS-CoV-2 induced acute anosmia and ageusia in golden Syrian hamsters, lasting as long as the virus remained in the olfactory epithelium and the olfactory bulb. Finally, olfactory mucosa sampling from patients showing long-term persistence of COVID-19-associated anosmia revealed the presence of virus transcripts and of SARS-CoV-2-infected cells, together with protracted inflammation. SARS-CoV-2 persistence and associated inflammation in the olfactory neuroepithelium may account for prolonged or relapsing symptoms of COVID-19, such as loss of smell, which should be considered for optimal medical management of this disease.

## Teaser

SARS-CoV-2 persists in the olfactory epithelium of hamsters and in individuals with COVID-19 for several months after infection.

## INTRODUCTION

COVID-19, caused by SARS-CoV-2 commonly induces airway and pulmonary symptoms, and in severe cases leads to respiratory distress and death (*1*). Although COVID-19 is primarily a respiratory disease, many patients exhibit extra-respiratory symptoms of various severity. Among these, a sudden loss of olfactory function in SARS-CoV-2-infected individuals was reported worldwide at the onset of the pandemic. Loss of smell (anosmia) and/or of taste (ageusia) are considered now as cardinal symptoms of COVID-19 (*2*–*4*). Likewise, a wide range of central and peripheral neurological manifestations have been observed in severe patients. Although neuropilin-1 was found to facilitate SARS-CoV-2 entry in neural cells (*5*), and thus a neurotropism of SARS-CoV-2 could be suspected, a direct role of the virus in the neurological manifestations remains highly debated (*2*, *6*).

The *bona fide* virus entry receptor is the angiotensin-converting enzyme 2 (ACE2), which is expressed along the entire human respiratory system, thereby accounting for SARS-CoV-2 respiratory tropism (*7*, *8*). In the upper airways, and more precisely in the superior-posterior portion of the nasal cavities resides the olfactory mucosa. This region is where the respiratory tract is in direct contact with the central nervous system (CNS), via olfactory sensory neurons (OSN), of which cilia emerge within the nasal cavity and their axons project into the olfactory bulb (*9*). As loss of smell is a hallmark of COVID-19, and several respiratory viruses (influenza, endemic human CoVs, SARS-CoV-1) invade the CNS through the olfactory mucosa via a retrograde route (*10*), we hypothesized that SARS-CoV-2 might be neurotropic and capable of invading the CNS through OSNs.

SARS-CoV-2 can infect neurons in human brain organoids (*11*) and recent reports have confirmed the presence of SARS-CoV-2 in olfactory mucosa OSNs that express neuropilin-1 (*5*) and deeper within the CNS at autopsy (*12*, *13*). Yet, the portal of entry of SARS-CoV-2 in the CNS remains elusive, as well as the exact mechanism leading to the olfactory dysfunction in COVID-19 patients. Various hypotheses have been proposed such as conductive loss due to obstruction of the olfactory cleft (*14*), alteration of OSN neurogenesis (*15*) and secondary CNS damage related to edema in the olfactory bulb (*16*, *17*). Detailed study of the olfactory system and olfaction in living patients with COVID-19 is thus needed to investigate the SARS-CoV-2 neuroinvasiveness in the olfactory neuroepithelium.

Complementary to this approach, animal models recapitulating the biological and clinical characteristics of SARS-CoV-2-related anosmia and ageusia would constitute useful tools to address deeper mechanisms. In this regard, wild-type mice are poorly susceptible to SARS-CoV-2 infection as the mouse ACE2 ortholog is not acting as a receptor for this virus (*18*), and the various transgenic mouse lines expressing the human version of the virus entry receptor (hACE2) under the control of different promoters, display disproportionate high CNS infection leading to fatal encephalitis (*19*–*22*), which rarely occurs in patients with COVID-19. This mismatch likely reflects the artefactual ectopic and high expression of hACE2 caused by the different transgene promoters. In contrast, the golden Syrian hamster (*Mesocricetus auratus*) expresses an endogenous ACE2 protein able to interact with SARS-CoV-2 (*18*) and constitutes a naturally-permissive model of SARS-CoV-2 infection (*23*–*25*). Previous reports have shown infection in hamster olfactory mucosa, but whether olfactory neurons can be infected or only non-neuronal, epithelial sustentacular cells, remains controversial (*26*, *27*). Moreover, the link between infection, inflammation and tissue disruption of the olfactory neuroepithelium and corresponding brain regions is unclear. Likewise, how damage of the neuroepithelium correlates with anosmia, and the potential SARS-CoV-2 neuroinvasion from the olfactory system to its downstream brain structures, remains highly debated.

Here, we report the interactions of SARS-CoV-2 with the olfactory system and its pathophysiological mechanisms. We first investigated SARS-CoV-2 infection of the olfactory mucosa in patients with COVID-19, and recent loss of smell. Because olfactory mucosa biopsy is an invasive procedure, which cannot be used for research purpose in patients with COVID-19, we performed nasal mucosa brush sampling, a non-invasive technique previously used in patients to study neurodegenerative and infectious diseases (*28*–*30*). We next attempted to model SARS-CoV-2-associated anosmia/ageusia in golden Syrian hamsters to further investigate the pathogenesis of neuroepithelium and CNS infection. Finally, we investigated the olfactory mucosa of post-COVID-19 patients presenting long-lasting olfactory dysfunction.

## RESULTS

### SARS-CoV-2 detection in the olfactory mucosa of patients with COVID-19 exhibiting loss of smell

We enrolled 7 patients that were referred to the ear, nose and throat (ENT) department for olfactory function loss and COVID-19 suspicion in the context of the COVID-19 first wave in Paris, France, alongside with 4 healthy controls. The main clinical features of patients and controls are listed in Tables S1 and S2. The time from first COVID-19-related symptoms to inclusion in the study ranged from 0 to 13 days. None of the patients required hospitalization. Their prominent symptom was recent loss of olfactory function (sudden for 6 patients but progressive for case #1) and was accompanied with taste changes (except case #3) and at least one symptom belonging to the clinical spectrum of COVID-19, such as diarrhea, cough, dyspnea, conjunctivitis, fever, fatigue, headache, muscle pain, laryngitis or a sore throat (Fig. S1A). Olfactory function loss was the first symptom related to COVID-19 in cases #5 and #14 whereas it was preceded by, or concomitant to other symptoms in the remaining patients. Smell loss was deemed severe for cases #1, #2, #4, #5, #14 and #15, and moderate for case #3. Taste loss was deemed severe for cases #1, #2, #4, #5, and #14 and mild for case #15. The characteristics of the taste and smell abnormalities are listed in Table S3. Other otolaryngologic symptoms were rhinorrhea for 4 patients, not concomitant with smell loss, nasal irritation for 2 patients and hyperacusis for case #1. Nasal obstruction was not reported in any of the patients. Taste changes were characterized in the 6 patients by dysgeusia where they had a reduced acuity for sweet taste, had a bad taste in the mouth, reduced or increased acuity for bitter, reduced acuity for salt or sour were reported in 4 out of the 6 patients with dysgeusia. Three patients (#2, #4 and #15) were unable to discriminate between different food flavors such as meat and fish.

To investigate whether infection in the olfactory mucosa was associated with olfactory functional loss, all patients underwent olfactory mucosa brush cytological sampling. Four patients had detectable SARS-CoV-2 RNA, using the conventional nasopharyngeal samples at inclusion (Tables S1 and S2). All patients, but none of the controls, had detectable SARS-CoV-2 RNA in cytological samples from the olfactory mucosa using the RT-qPCR SYBR green technique, unambiguously confirming the diagnosis of SARS-CoV-2 infection (Tables S1 and S2). To further investigate if the presence of viral RNA in the olfactory mucosa reflected active replication of SARS-CoV-2 genome, we performed a comparative analysis of genomic and subgenomic copy numbers by RT-qPCR. Patients #2, #14 and #15 exhibited a strong viral genomic RNA load in the olfactory mucosa (2.25. 10^6^ RNA copies/μL, 8.09. 10^7^ RNA copies/μL, 7.17. 10^6^ RNA copies/μL, respectively), and subgenomic RNA was detected in patient #14 (5.66. 10^6^ copies/μL), whereas other cases were detected as positive (above the limit of detection with RT-qPCR SYBR green) but not quantifiable (below the limit of quantification, less than 200 RNA copies/μL using the RT-qPCR Taqman technique) (Tables S1 and S2). Given the high viral genomic RNA load in the olfactory mucosa of the patients #14 and #15, we determined the SARS-CoV-2 titer in their olfactory mucosa. Infectious SARS-CoV-2 were isolated from the olfactory mucosa of patients #14 (1.19×10^6^ PFU/mL) and #15 (5.38×10^2^ PFU/mL) but not in sex- and age-matched controls, indicating that infectious virus is indeed present in the olfactory mucosa of anosmic patients (Table S2).

**Fig. 1:**
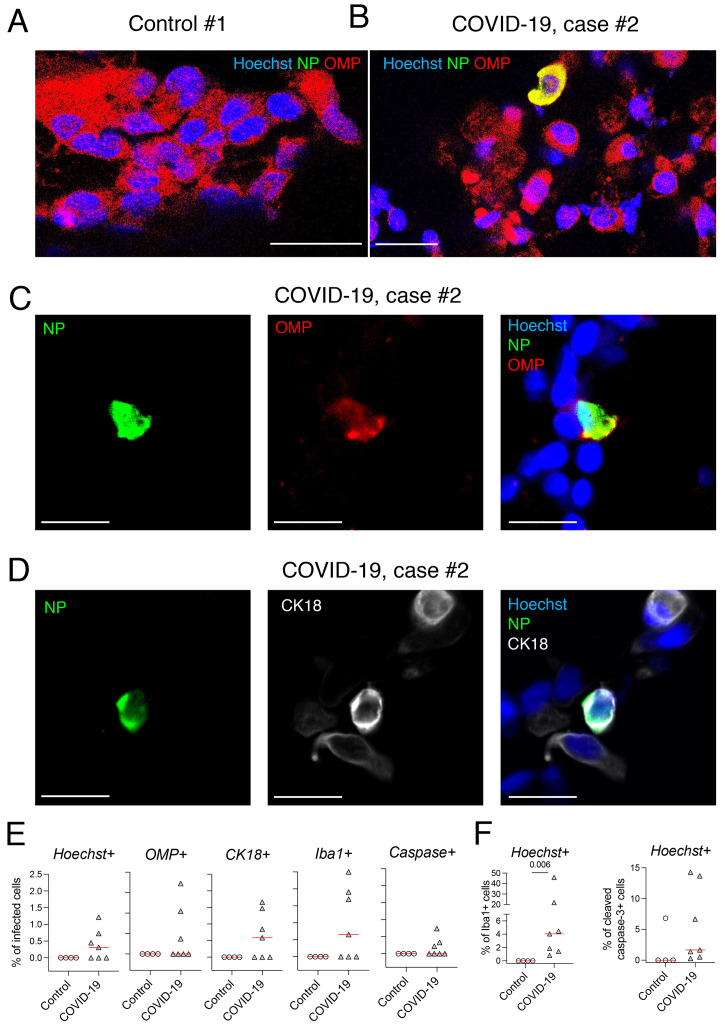
**Analysis of olfactory mucosa from patients with COVID-19 with acute olfactory function loss, at early stage of infection**. (**A**) Immunofluorescence of cells retrieved from the olfactory mucosa of the control subject #1. (**B**) Cells retrieved from the olfactory mucosa of the COVID-19 patient #2. (**C, D**) Close-up immunofluorescence images of olfactory epithelium samples from COVID-19 patient #2. Infected mature olfactory neurons (OMP^+^) are observed (**B, C**), alongside sustentacular CK18^+^ cells (**D**). (**E**) Percent of infected NP+ cells among: Hoechst+ cells, OMP+ cells, CK18+ cells, Iba1+ cells and cleaved caspase 3+ cells. (**F**) Percent of Iba1+ cells among Hoechst+ cells (left), and percent of cleaved caspase 3 + cells among Hoechst+ cells (right). SARS-CoV-2 is detected by antibodies raised against the viral nucleoprotein (NP). N=4 controls, N=7 patients with COVID-19 (E, F); Horizontal red lines indicate the medians. Mann-Whitney test (E, F); The p value is indicated when significant. Scale bars = 20μm (**A, B**) or 10μm (**C, D**).

**Fig. 2:**
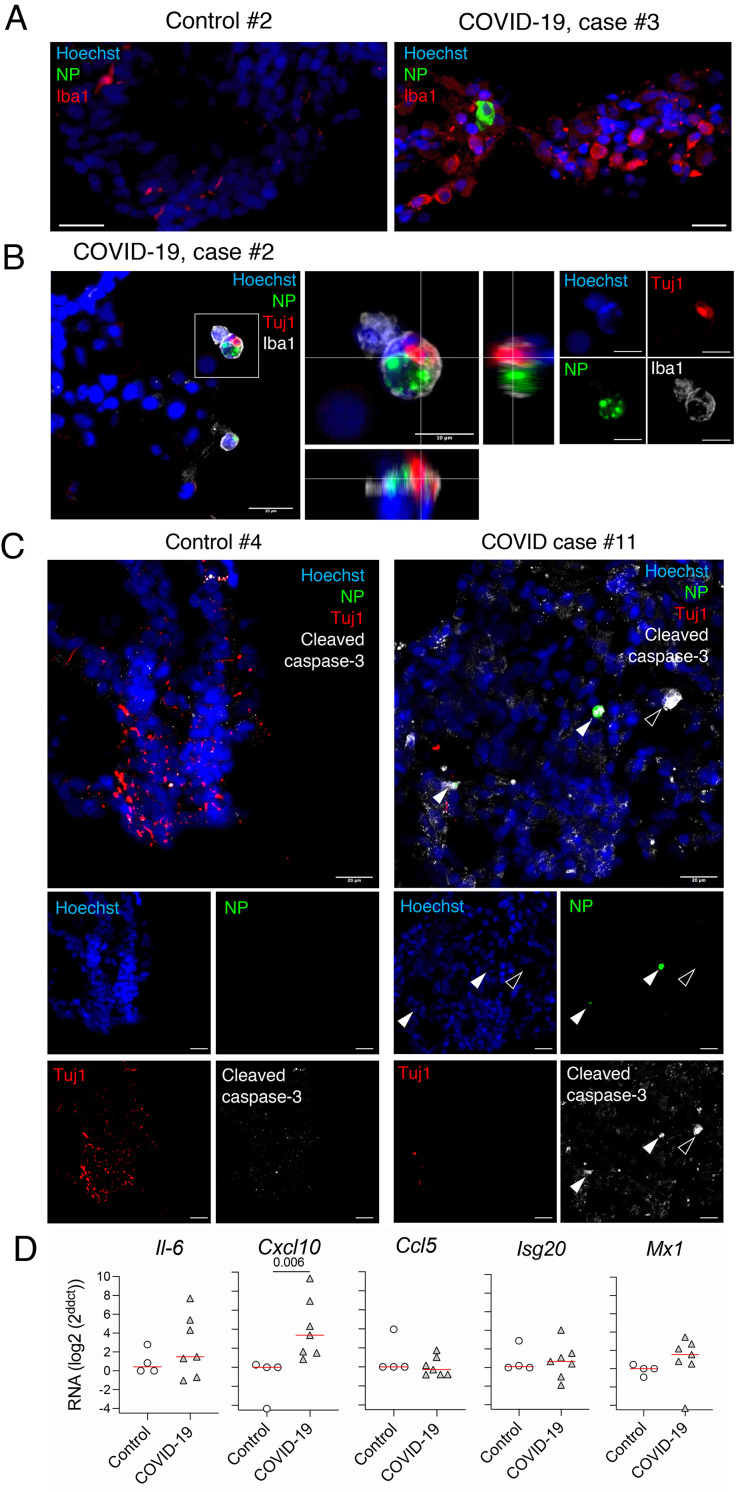
**Immune response in olfactory mucosa of patients with COVID-19 with acute olfactory function loss**. (**A**) Immunofluorescence of myeloid cells (Iba1+) retrieved from the olfactory mucosa of the control subject #2 vs COVID-19 case #3. (**B**) Orthogonal projection of Tuj1+ antigens included in infected Iba1+ cell. (**C**) Immunofluorescence of apoptotic cells (cleaved caspase-3+) of control #4 and COVID-19 case#11. Solid arrows: infected cells positive for cleaved caspase-3. Empty arrow: non-infected cell positive for cleaved caspase-3. (**D**) Cytokines and chemokines transcripts in the olfactory mucosa. N=4 controls, N=7 patients with COVID-19. Horizontal red lines indicate the medians. Mann-Whitney test (D). The p value is indicated when significant. Scale bars = 20μm or 10μm (B, inset).

We further investigated the viral presence in the olfactory mucosa by immunofluorescence labeling of the cytological samples. Variable cell density between olfactory mucosa samples from the COVID-19 and control individuals was found, but all samples contained mature OSNs. Indeed, cells positive for OMP and PGP9.5 were consistently present, and *OMP* transcripts recovered from all the samples, validating the quality of the swabbing procedure (Fig. 1A and B; Fig. S1B and Tables S1 and S2). Immunostaining revealed the presence of SARS-CoV-2 antigens (nucleoprotein, NP) in 4 patients (RT-qPCR+) out of 7 but never in controls (Tables S1 and S2, Fig. 1). We observed numerous Iba1^+^ (immune myeloid) cells in the olfactory mucosa of all patients whereas few to no Iba1^+^ cells in controls (Fig. 1E and 2A, Tables S1 and S2,). These data suggest that SARS-CoV-2 infection is associated with inflammation of the olfactory mucosa in patients with olfactory impairment, we thus measured the profile of local cytokine and inflammatory mediators. Expression of gene transcript of *Cxcl10* was elevated in the olfactory mucosa in most patients with detectable SARS-CoV-2 antigens as compared to control patients, and in contrast, an interindividual variability, both in SARS-CoV-2-infected and control individuals, was observed in *Il-6*, *Ccl5*, *Isg20* and *Mx1* gene transcript expression (Fig. 2D, Tables S1 and S2).

Together, this first set of data indicates that SARS-CoV-2 exhibits an unambiguous tropism for the olfactory neuroepithelium, and this infection is associated with increased local inflammation. We next investigated the identity of the cell types targeted by SARS-CoV-2. We detected SARS-CoV-2-infected mature sensory neurons (OMP^+^; Fig. 1, B, C and E, Tables S1 and S2); other SARS-CoV-2 infected cells were sustentacular cells (expressing CK18, Fig. 1D and E, and Fig. S1C), and myeloid cells (expressing Iba1, Fig. 1E and 2B, Tables S1 and S2). We also detected the presence of immature sensory neurons (Tuj-1^+^) in the olfactory mucosa of all patients, some of them being infected by SARS-CoV-2. Some Iba1 and SARS-CoV-2 positive cells were engulfing portions of Tuj-1 cells in the olfactory mucosa of COVID case #2, suggesting that infected immature sensory neurons were in the process of being phagocytosed by innate immune cells (Fig. 2B). We next investigated whether infection induces cell death in the olfactory neuroepithelium, by cleaved caspase-3 staining. A strong cleaved caspase-3 signal was detected both in infected and non-infected cells in patients with COVID-19, whereas no signal was detected in samples from control individuals (Fig. 1, E and F, Fig. 2C, Tables S1 and S2). Altogether these results show that a variety of cell types are infected in the olfactory neuroepithelium of patients with COVID-19, leading to increased cell death through apoptosis. Among them, the loss of mature OSN might be critically relevant in the context of the anosmia. To further assess the impact of neuroepithelium infection by SARS-CoV-2, we infected Syrian golden hamsters to experimentally reproduce anosmia and ageusia, and investigated the potential SARS-CoV-2 infection of the olfactory system and its upstream brain structures.

### Modeling loss of taste and smell functions using SARS-CoV-2 nasal instillation in golden hamsters

**Fig. 3:**
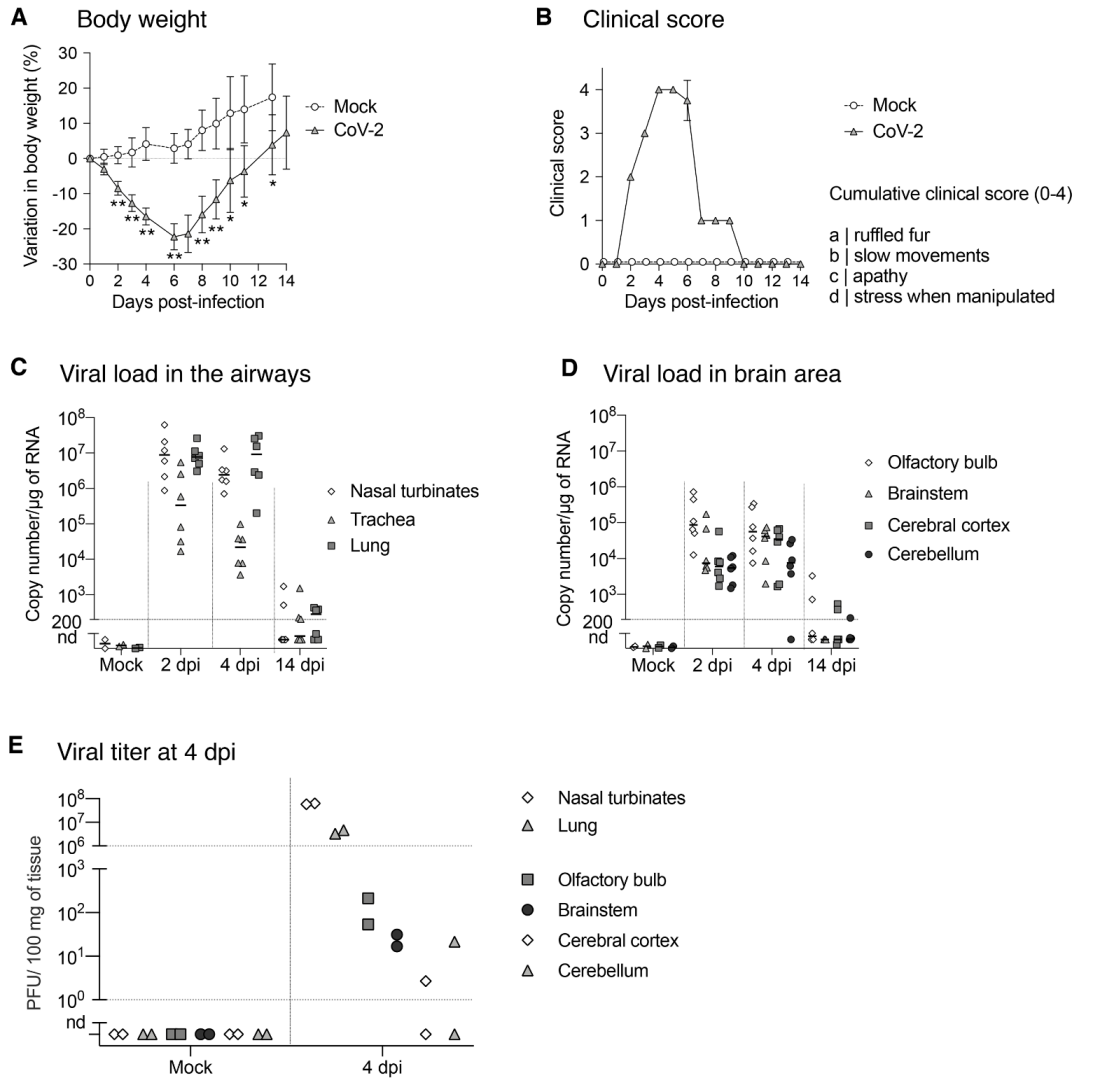
Clinical and molecular characteristics of experimental infection with SARS-CoV-2 in golden hamsters. (**A-B**) Variation in body weight (**A**) and clinical score (**B**) of mock- and SARS-CoV-2 infected hamsters for 14 days post-infection (dpi). (**C, D**) Quantification of SARS-CoV-2 RNA in hamster airways (**C**) and in different brain areas (**D**) of mock and infected-animals at 2, 4 and 14 dpi. (**E**) Infectious viral titer in the nasal turbinates, lung, olfactory bulb, brainstem, cerebral cortex and cerebellum at 4 dpi expressed as Plaque Forming Units (PFU)/100 mg of tissue. Horizontal lines indicate medians. N=4-8/ timepoint in (**A, B**); N=6/timepoint in (**C, D**); N=2/timepoint in (**E**). Mann-Whitney test comparing infected animals to mock (A). *p<0.05; **p<0.01.

Syrian golden hamsters (both sexes) were intranasally inoculated with 6.10^4^ PFU of SARS-CoV-2 and followed-up between 24h and 14 days post-inoculation (dpi). Clinical, sensorial and behavioral functions were assessed at different timepoints (Fig. S2A). SARS-CoV-2 inoculation resulted in a decrease in body weight and a degradation in the clinical score as early as 2 dpi, with a peak between 4 and 6 dpi, and sickness resolution by 14 dpi (Fig. 3, A and B). High viral loads were detected throughout the airways of infected hamsters at 2 and 4 dpi and remained detectable even at 14 dpi (Fig. 3C) consistent with the well-established respiratory tropism of SARS-CoV-2. In line with our observations in human samples, the nasal turbinates of infected hamsters exhibited high viral loads as soon as 2 dpi. Viral RNA was also detected from 2 dpi and onward in various parts of the brain, including the olfactory bulb, cerebral cortex, brainstem (diencephalon, midbrain, pons and medulla oblongata) and cerebellum (Fig. 3D). Additionally, we were able to isolate infectious viral particles from the nasal turbinates, the lung and different brain areas (olfactory bulb, cerebral cortex, brainstem and cerebellum), indicative of the replication and production of SARS-CoV-2 in the CNS of hamsters (Fig. 3E). Having shown the concomitant infection of nasal turbinates and the CNS, we further investigated their impact on sensory and behavioral responses.

**Fig. 4:**
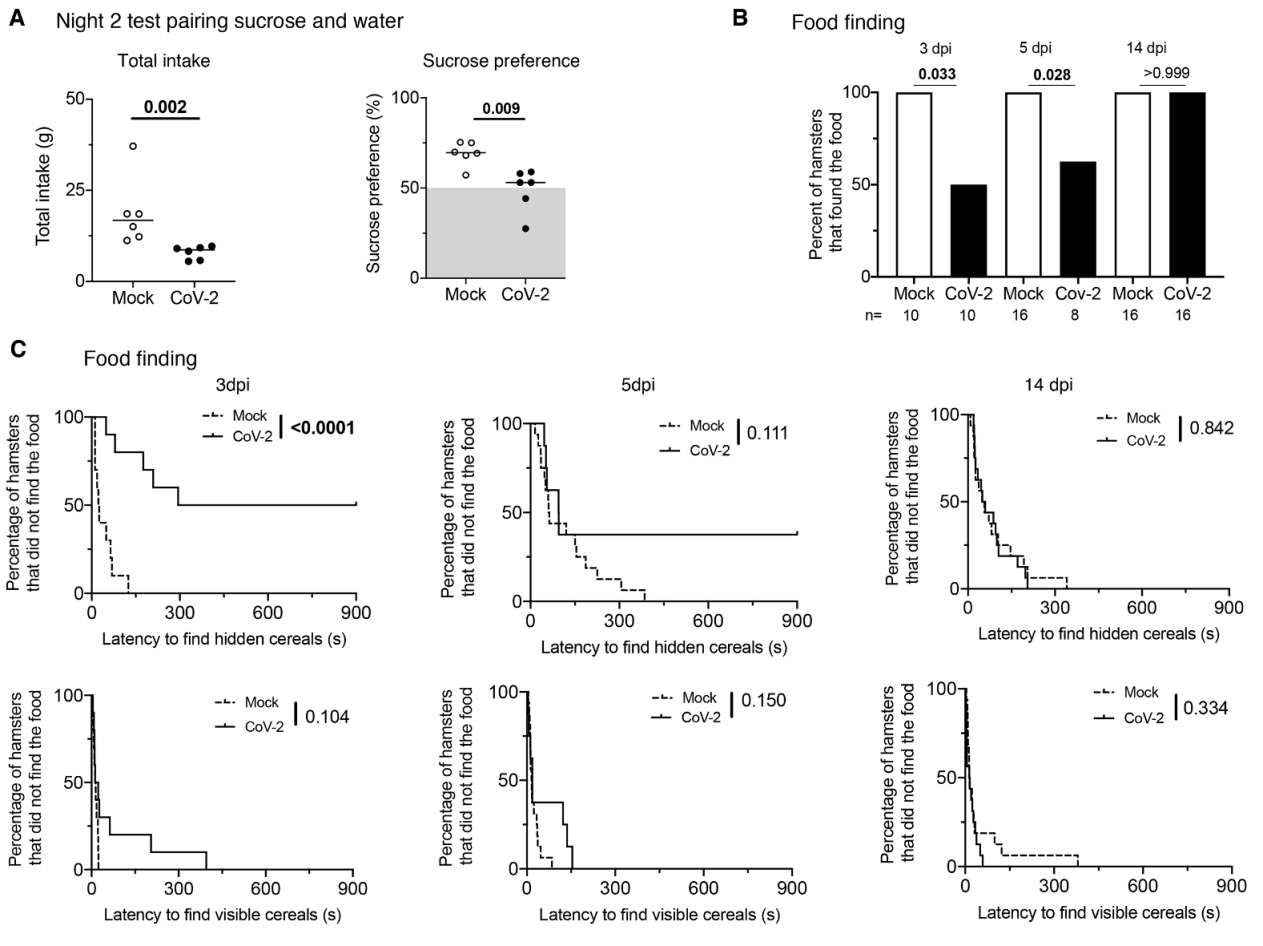
**Experimental infection with SARS-CoV-2 in golden hamster induces transient anosmia and ageusia**. (**A**) Variation in total consumption of liquid overnight and preference toward 2% sucrose-containing water of control and SARS-CoV-2 infected hamsters at 2 dpi. (**B**) Fraction of control or infected hamsters successfully finding hidden food in 15 min. (**C**) Fraction of control or infected hamsters successfully finding hidden or visible food over time. Food-finding assays were performed at 3, 5- and 14-dpi. Mann-Whitney test (A), Fisher’s exact test (B) and Log-rank (Matel-Cox) test (C). P value is indicated in bold when significant. Bars indicate medians. N=6-per group in (**A**); N=10 mock and N=10 CoV-2 at 3 dpi, N=16 mock and N=8 CoV-2 at 5 dpi, N=16 per group at 14 dpi in (**B, C**).

We assessed both gustatory and olfactory function of SARS-CoV-2-inoculated hamsters. At 2 dpi, we subjected hamsters to a sucrose preference test. As expected, mock-infected animals displayed a clear preference toward sucrose-complemented water compared to control water, whereas infected hamsters had no preference toward the sucrose-complemented water (Fig. 4A), indicative of a SARS-CoV-2-associated dysgeusia/ageusia. Moreover, infected animals exhibited signs of hyposmia/anosmia during food findings experiments, as they needed more time to find hidden (buried) food than uninfected hamsters, and a substantial proportion of them (50% at 3dpi and 37.5% at 5 dpi) failed to find the food at the end of the test (Fig. 4 B and C). Nevertheless, all infected hamsters succeeded to find visible food (Fig. 4C) revealing that no sickness behavior, visual impairment or locomotor deficit accounted for the delay in finding the hidden food. Also, no locomotor deficit was observed either during the open field (Fig. S2B) or painted footprint tests (Fig. S2C), further excluding a motor deficit bias during the food finding test. At 14 dpi, when weight and clinical score had resumed to normal (Fig. 3A and B), all hamsters successfully found the hidden food, indicating that infection-associated anosmia recovered spontaneously in this animal model.

### SARS-CoV-2 promotes cellular damages in the olfactory epithelium of infected hamsters

**Fig. 5:**
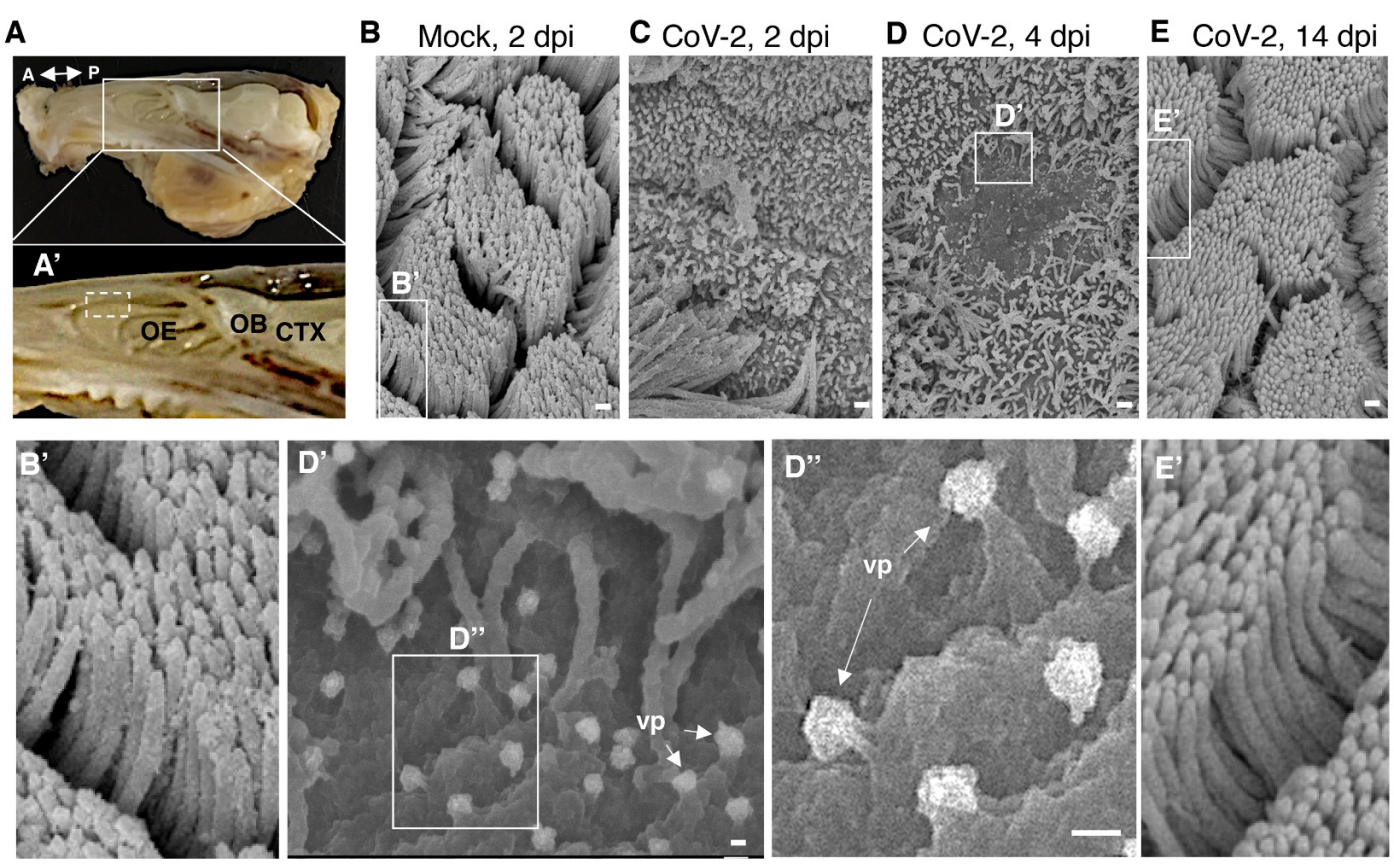
**SARS-CoV-2 induces loss of ciliation in the olfactory epithelium**. (**A**) Dissected hamster head, skin and lower jaw removed, sagitally cut in half. Double-headed arrow denotes the antero-posterior (A-P) axis. Close-up in **A’** shows the close relationship between the olfactory epithelium (OE), the olfactory bulb (OB) and the cerebral cortex (CTX). Discontinuous square indicates the area collected for scanning electron microscopy. (**B-E**) Scanning electron microscope imaging showing changes in olfactory epithelium following SARS-CoV-2 infection. The olfactory epithelium of mock- (**B, B’**) and CoV-2 inoculated hamsters at 2 dpi (**C**), 4 dpi (**D, D’, D”**) and 14 dpi (**E, E’**). A loss of cilia is observed at 2 and 4 dpi for infected hamsters. Viral particles (vp) are seen emerging from deciliated cells (**D’-D’’**, white arrows). Scale bars: 1 μm (B-E), 100 nm (D’, D”).

We then investigated the impact of SARS-CoV-2 infection on hamster olfactory mucosa, which exhibited high viral loads (Fig. 3C). The uppermost part of nasal turbinates is overlaid by the olfactory neuroepithelium (Fig. 5A), a neuroepithelium composed of sensory neurons and support sustentacular cells with both cell populations being ciliated. Imaging by scanning electron microscopy of the olfactory neuroepithelium showed an important loss of ciliation as early as 2 dpi (Fig. 5 B and C, Fig. S3) on large portions of the epithelial surface, indicating cilia loss in both OSNs and sustentacular cells. At 4 dpi, viral particles were seen budding from deciliated cells (Fig. 5D). At 14 dpi, the olfactory mucosa appeared ciliated anew, indistinguishable from that of mock-infected animals (Fig. 5E and fig. S3), consistent with the recovery of olfaction seen in infected hamsters (Fig. 4C).

**Fig. 6:**
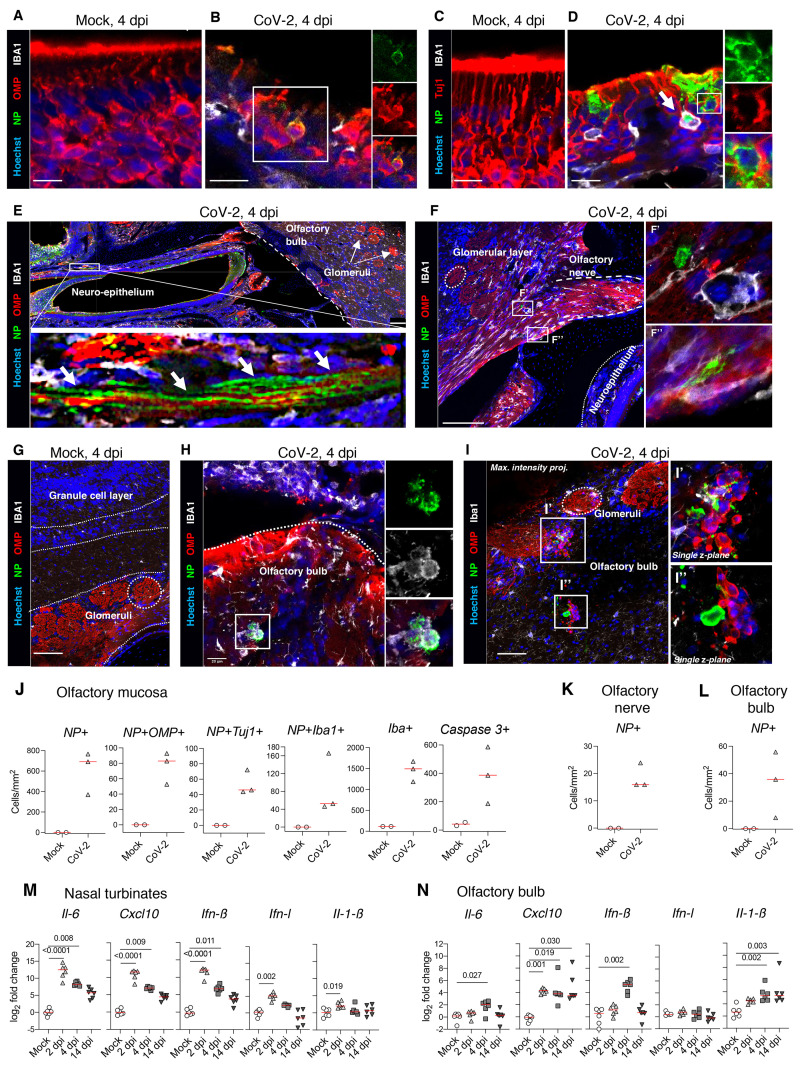
SARS-CoV-2 antigens detection and cytokine/chemokine transcripts quantification in the olfactory system of hamsters. (**A-D**) Olfactory epithelium of mock- (**A, C**) and SARS-CoV-2 (**B, D**) infected hamsters at 4 dpi. Insets show infected OMP^+^ mature olfactory sensory neurons (**B**), or infected Tuj1^+^ immature olfactory sensory neurons (**D**). The arrow in D indicates an infected Iba1+ cell. (**E**) Sagittal section showing nasal turbinates and olfactory bulb of SARS-CoV-2 infected hamster at 4 dpi. Inset depicts SARS-CoV-2 staining in olfactory sensory neuron axons. (**F**) Olfactory sensory axons projecting into glomeruli in the olfactory bulb of SARS-CoV-2 inoculated hamsters at 4 dpi. Insets (**F’, F’’**) show infected cells. (**G-I**) Olfactory bulb of mock-(**G**) or SARS-CoV-2 (**H, I**) infected hamsters at 4 dpi. Iba1^+^ infected cells are shown in (**H**) and several infected cells are observed in (**I**). SARS-CoV-2 is detected by antibodies raised against the viral nucleoprotein (NP). Scale bars: 20μm (A-D, H), 100μm (E, F), 50μm (G, I). Images are single z-planes (A-H) or maximum intensity projection over a 6μm depth (I). (**J**) Number of NP+ cells, NP+OMP+ cells, NP+Tuj1+ cells, NP+Iba1+, Iba1+ cells and cleaved caspase 3+ cells in the olfactory mucosa. (**K, L**) Number of NP+ cells in the olfactory nerve (K) and the olfactory bulb (L). (**M, N**) Cytokines and chemokines transcripts in the nasal turbinates (**M**) and in the olfactory bulb (**N**) at 2, 4 and 14 dpi. Mann-Whitney test (E, F). Kruskal-Wallis followed by the Dunn’s multiple comparison test (M, N). The p value is indicated when significant. Horizontal red lines indicate the medians.

In line with the detection of viral particles by electron microscopy at 4 dpi, SARS-CoV-2 was detected in the hamsters’ olfactory mucosa at this time point, alongside an infiltration of myeloid Iba1^+^ cells (Fig. 6 A-E and J). In the olfactory mucosa, SARS-CoV-2 antigens were found in the cytoplasm of mature (OMP^+^; Fig. 6A, B and J) and immature (Tuj1^+^; Fig. 6C, D and J) sensory neurons and in sustentacular cells (CK18^+^; Fig. S4A and B). Some Iba1^+^ immune cells seen infiltrating the neuroepithelium were positive for SARS-CoV-2, consistent with a potential secondary infection resulting from the phagocytosis of infected cells (Fig. 6D, arrow and Fig. 6J). Of note, the areas of neuroepithelium containing infected cells were disorganized (see Fig. 6B and D, and Fig. S4B), whereas adjacent areas without SARS-CoV-2 remained morphologically intact (Fig. S4C). Cilia of OMP^+^ neurons located at the apical part of olfactory epithelium were lost in the disorganized infected neuroepithelium (Fig. 4B). As observed in human samples (see Fig. 2C), infection induced cell death, with many neuronal and non-neuronal cells being positive for cleaved-caspase-3 in the olfactory mucosa of infected hamsters at 4 dpi (Fig. 6J, Fig. S4D). Importantly, we observed the expression of cleaved caspase-3 in infected as well as uninfected cells (Fig. S4D, indicating that cell death is not only caused by cytopathic effects of SARS-CoV-2, but also probably by the local inflammation and immune responses to infection.

### SARS-CoV-2 dissemination to the brain and neuroinflammation in infected hamsters

Having shown that SARS-CoV-2 infects OSNs, and that SARS-CoV-2-infected hamsters exhibit signs of anosmia and ageusia, we wondered whether SARS-CoV-2 invades the CNS via a retrograde route from the olfactory system. SARS-CoV-2 was detected in olfactory nerve bundles close to the neuroepithelium, as demonstrated by the co-localization of SARS-CoV-2 nucleoprotein antigen and OMP^+^ sensory neuron axons reaching the olfactory bulb (Fig. 6E, K and L), consistent with a retrograde infection of axons. Furthermore, SARS-CoV-2 nucleoprotein was detected at the junction of the olfactory nerve and olfactory bulb, seemingly infecting cells of neuronal/glial morphology (Fig. 6F). In the olfactory bulb, SARS-CoV-2 nucleoprotein was detected in Iba1^+^ cells (Fig. 6H) and in uncharacterized cells (Fig. 6I and L) in the glomerular layer of the olfactory bulb. The viral nucleoprotein was not detected in other areas of the brain. The high viral RNA loads in the nasal turbinates and in the olfactory bulb, together with the observation of viral antigens along the entire route from the olfactory sensory organ to the bulb, suggests that SARS-CoV-2 enters the brain through the olfactory system, although this finding does not rule out other port of central nervous system entry in patients with COVID-19.

In the nasal turbinates, we detected an intense pro-inflammatory environment, with an up-regulation of *Il-6*, *Cxcl10*, *Ifn-β, Ifn-λ* and *Il-1β* at 2 dpi, and a slight decrease at 4 and 14 dpi (Fig. 6M). Similarly, the olfactory bulb exhibited an important up-regulation in the expression of these genes (Fig. 6N), but in a different and delayed pattern compared to the nasal turbinates: whereas *Cxcl10* was overexpressed throughout the infection, there was no change in *Ifn-λ,* and the increase in *Il-6*, *Ifn-β* and *Il-1β* gene expression was observed only from 4 dpi, with *Il-1β* remaining up-regulated up to 14 dpi. These data reveal bulbar inflammation during the SARS-CoV-2 infection, possibly in response to signaling via olfactory nerves.

**Fig. 7:**
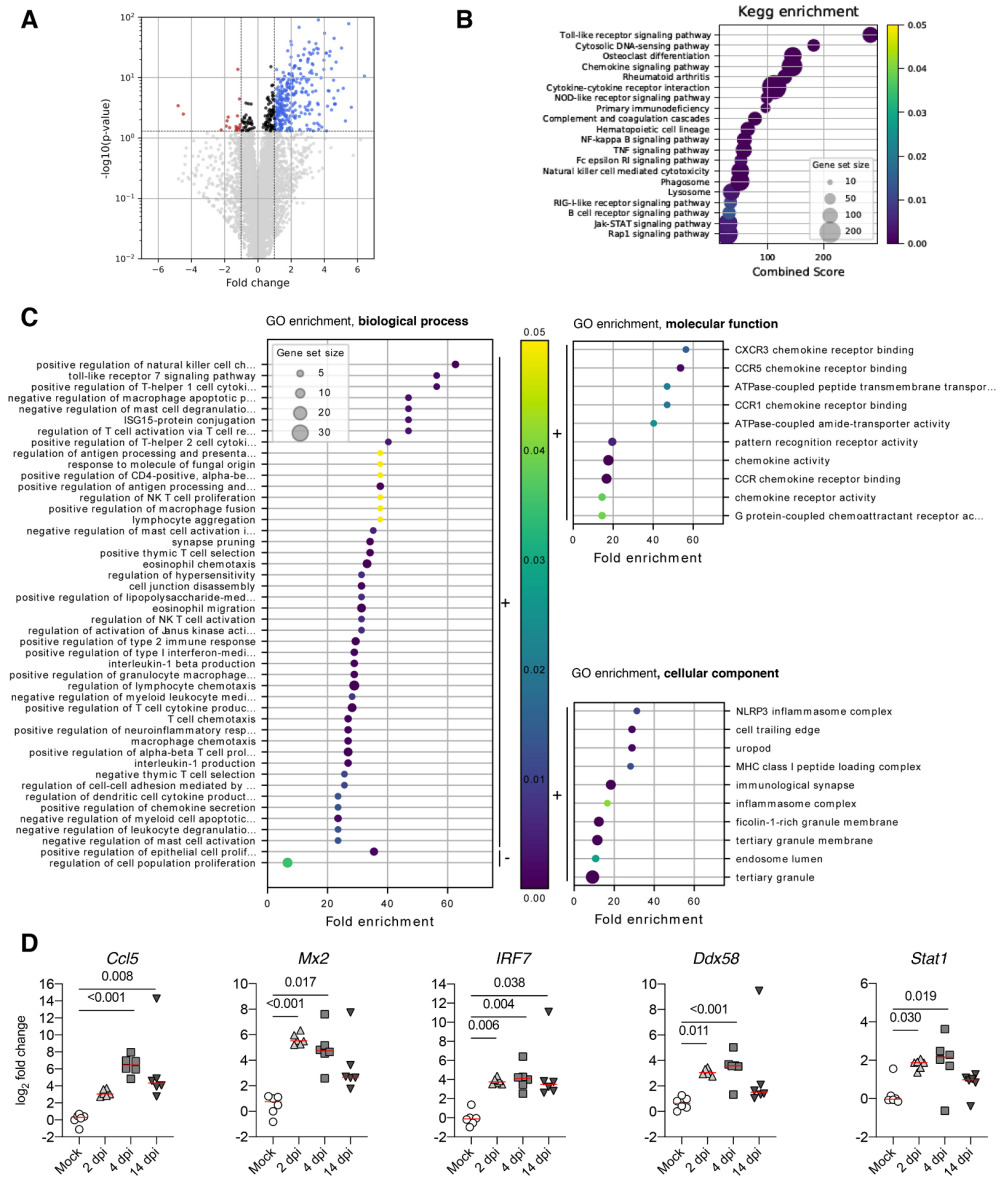
Differentially expressed genes in the olfactory bulb of golden hamsters infected by SARS-CoV-2 (at 4 dpi) derived by RNA-seq. (**A**). Volcano plot of the comparisons between infected and non-infected samples. Y-axis represents the Benjamini-Hochberg corrected p-value on a logarithmic scale (-log_10_). Grey dots represent genes not passing a threshold of FDR < 0.05. Black dots represent genes passing the FDR threshold but having fold changes between -1 and 1. Red and blue dots correspond to significant down and up-regulated genes with a fold change inferior to -1 or superior to 1, respectively. (**B**). KEGG-pathways enrichment based on the differentially regulated genes between infected and non-infected samples. Only the 20 highest combined scores are plotted. Circle sizes are proportional to the gene set size. Circle color is proportional to the corrected p-values and corresponds to the scale presented in C, D and E. (**C**). GO enrichment analysis considering biological process only. Selected GO terms are based on the up and down-regulated genes between infected and non-infected samples. The black bars on the right-hand side of the scatter plot indicate enrichment based on down (“-”) and up (“+”) regulated gene sets. Only the 50 highest fold enrichments are plotted for the up regulated gene set. Circle sizes are proportional to the gene set size, which shows the total size of the gene set associated with GO terms. Circle color is proportional to the corrected p-values and corresponds to the scale presented between C, D and E. GO enrichment analysis considering molecular function and cellular components related Figures follow the same construction as in biological process, with the exception that only the 10 highest fold enrichments are plotted for the up regulated gene set. (**D**) Validation targets in the olfactory bulb at 2, 4 and 14 dpi. n=6/time-point. Kruskal-Wallis followed by the Dunn’s multiple comparison test (J, K). The p value is indicated when significant. Horizontal lines indicate the medians. Complete analysis is listed in Supplementary Data file S2

Using RNA-seq, we observed 374 and 51 differentially expressed genes (DEG; increased or decreased, respectively) in the olfactory bulbs of SARS-CoV-2 infected hamsters at 4 dpi (Fig. 7A). The DEG were classified according to KEGG (Kyoto Encyclopedia of Genes and Genomes) pathways (Fig. 7B) and the GO (gene ontology) terms based on their biological processes, molecular functions and cellular components (Fig. 7C). Up-regulated genes were mainly involved in inflammatory responses and responses to virus infection, with innate immunity components (type-I IFN-mediated response, NK cell activation, TLRs, RLRs, NF-κB and Jak-STAT signaling pathways), adaptive immunity components (T_H_1, T_H_2, CD4^+^ T-cells) and functions related to chemokine signaling. Other biological processes related to nervous system functions were synapse pruning, up-regulation of the neuroinflammatory response, and both astrocyte and microglial activation. To validate the involvement of these signaling pathways, we analyzed the expression of selected targets in the olfactory bulb by RT-qPCR (Fig. 7D). The genes *Mx2*, *Irf7*, *Ddx58* and *Stat1*gene transcripts were found up-regulated early in the infection (2 and 4 dpi), whereas *Ccl5* was up-regulated only at 4 dpi. The overexpression of *Ccl5* and *Irf7* persisted even at 14 dpi. Altogether, SARS-CoV-2-associated inflammation in the bulb confirmed by unbiased RNA-seq analysis, along with the increased viral load detected in the brain parenchyma, supports the assumption that SARS-CoV-2 neuroinvasion drives the neuroinflammation. Of note, *Cxcl10*, *Il-1β, Ccl5* and *Irf7* overexpression persisted up to 14 dpi, when animals had recovered from ageusia/anosmia. These data indicate that an infectious or post-infectious inflammatory process persist even in the asymptomatic, or in a delayed post-symptomatic phase, in our animal model.

### SARS-CoV-2 persistence in human olfactory mucosa with long-lasting/relapsing loss of smell

In some patients, neurological impairments and/or sensory dysfunctions persist several months later from the onset of COVID-19 symptoms, and it has been proposed that this may be linked to persistent viral infection and/or inflammation (*31*, *32*). We recruited 4 patients with prolonged/recurrent olfactory function loss after COVID-19. The main characteristics of these patients are listed in Table S4 They were recruited between July 15 and 29, 2020, at a time where viral circulation in Paris was very low (<10 cases/100,000 inhabitants/week), implying that SARS-CoV-2 reinfection of these patients was very unlikely. In this case, the time from first COVID-19 related symptoms to inclusion ranged from 110 to 196 days. As a control, we sought to include patients with confirmed COVID-19 but without loss of smell. However, given the relative invasiveness of the olfactory epithelium brushing, the sampling of SARS-CoV-2 infected patients with neither loss of smell nor ENT care was not possible, except for a single individual (47 years old man, recruited in July 2020) presenting long-lasting COVID-19 symptoms (such as dysgeusia, vertigo, paresthesia and tremor, tingling of the face, nose, arms and legs, persisting for >141d), but without any history of anosmia (Table S4).

**Fig. 8:**
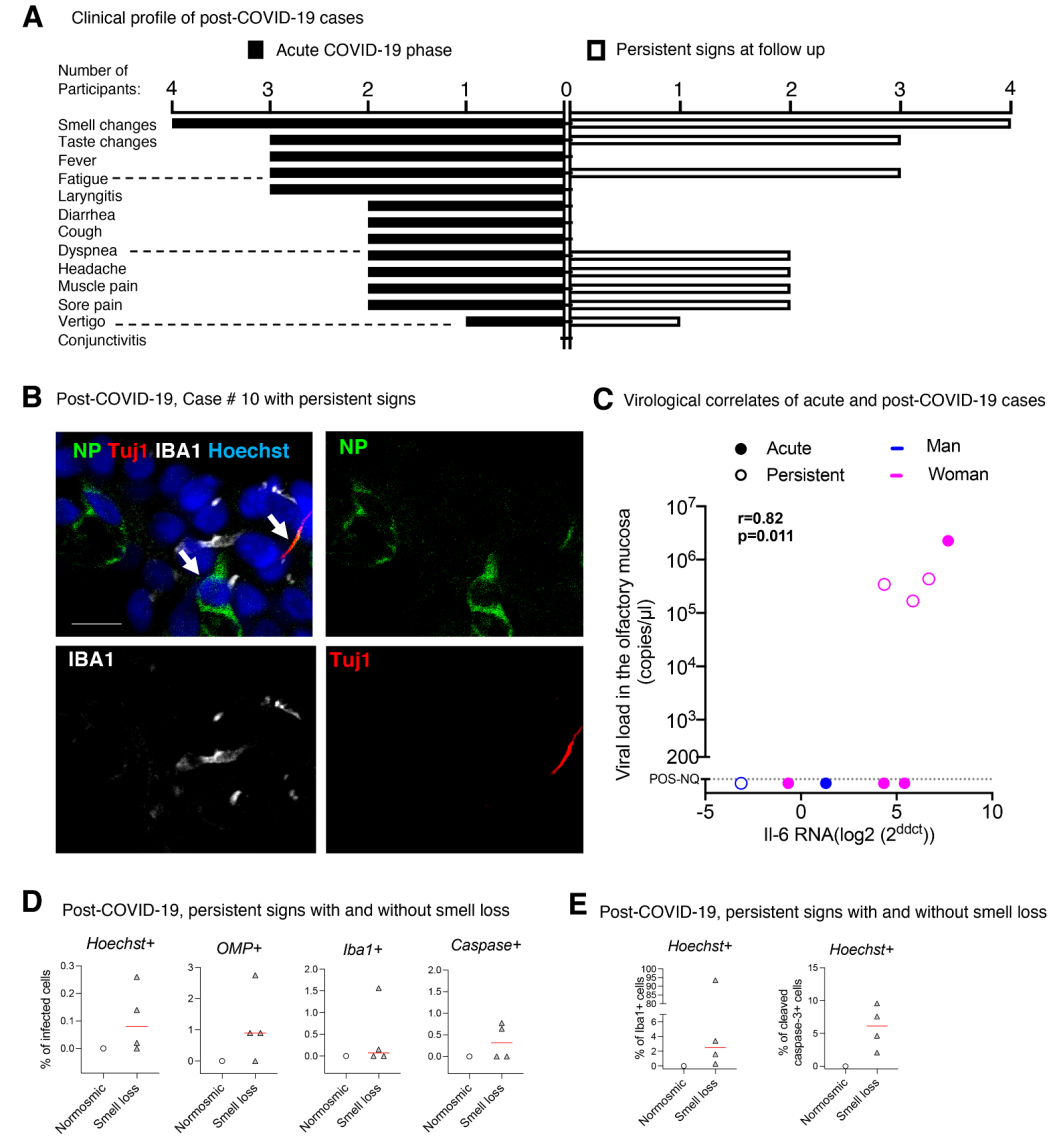
**SARS-CoV-2 is present in the olfactory mucosa from patients with persistent loss of smell post-COVID-19**. (**A**) Clinical profile of the 4 patients with prolonged loss of smell post-COVID-19. The general symptoms at the acute phase and at the follow up (inclusion in CovidSmell study) are shown. (**B**) Immunofluorescence of infected cells in the olfactory mucosa of the case #10 presenting with persistent olfactory dysfunction at 196 days after COVID-19 onset. The left arrow indicates an infected cell with viral NP staining. The right arrow indicates a Tuj1-NP co-labeling in another cell. (**C**) Graph depicting the correlation between the IL-6 mRNA expression and the viral load in the 9 patients with acute COVID-19 (“acute”: n=5) or persistent olfactory dysfunction post-COVID-19 (“persistent”, n=4). (**D, E**) Fraction of infected cells among: Hoechst+ cells, OMP+ cells, Iba1+ cells and cleaved caspase 3+ cells (D), and fraction of Iba1+ cells among Hoechst+ cells (left), and fraction of cleaved caspase 3 + cells among Hoechst+ cells (right) in the olfactory mucosa of patients with persistent loss of smell post COVID-19 (n=4) and the patient #7 (post-COVID-19, persistent signs without loss of smell). (**E**) Viral load values were assessed by Taqman qPCR; when not quantifiable (nq: <200 copies /μL), they were arbitrary given the 50 value for Spearman test (**C**). Variations in the cytokine gene expression were calculated as the n-fold change in expression in the swabs compared with the swab value of control #1 that was arbitrary put on zero value. Horizontal red lines indicate the medians (D, E). Mann-Whitney test D, E), Spearman test (**C**). Scale bar: 10 μm.

The four patients with long-lasting/relapsing loss of smell had been diagnosed with COVID-19 between January and March 2020, based on their initial clinical assessment, including sudden anosmia at disease onset, accompanied with taste changes (except case #8) and at least one clinical sign related to COVID-19, such as fever, fatigue, diarrhea, cough, dyspnea, headache, muscle pain, laryngitis, sore throat, but also paresthesia and vertigo in some patients (Fig. 8A). Smell loss was complete at disease onset for these patients. Other otolaryngologic symptoms were rhinorrhea for two patients, not concomitant with smell loss and nasal irritation for three patients. Nasal obstruction was reported in patient #10. All had persistent smell loss, persistent taste dysfunction (except case #8) and/or other neurological deficits after COVID-19 at inclusion (Fig. 8A) and were seen at the ENT department for this reason. Neurological signs were stereotypical crises of wriggling nose, left intercostal and non-specific arm pain (case #8), paresthesia (case #9) and vertigo (case #10). The characteristics of taste and smell abnormalities at inclusion are described in Table S5. Two patients complained of bad taste (Table S2). Reduced or increased acuity for bitter, reduced acuity for salt or sour were reported by the two patients with dysgeusia. None of the patients required hospitalization.

These four patients, when consulting with long-lasting/relapsing loss of smell, had no detectable SARS-CoV-2 RNA in nasopharyngeal samples by the mean of routine diagnosis RT-qPCR. However, all of them had detectable SARS-CoV-2 RNA in cytological samples from the olfactory mucosa, using the RT-qPCR SYBR technique (Table S4). Furthermore, three of them (#8, #9, #10) had a high viral genomic RNA load in the olfactory mucosa (1.68 to 4.35 10^5^ RNA copies/μL; Taqman technique), but no subgenomic RNA was detected (Table S4), in favor of a lack of active SARS-CoV-2 replication in the analyzed samples. We further evaluated olfactory mucosa infection by immunofluorescence labeling. We found variable cellularity between olfactory mucosa samples within patients, but all samples contained OSNs, positive for Tuj1, indicating the efficient sampling of the neuroepithelium. Immunostaining revealed the presence of SARS-CoV-2 antigens (N protein) in three out of four patients (Table S4, Fig. 8B and D). We observed abundant Iba1^+^ immune cells in the olfactory mucosa of all four patients (Table S4, Fig. 8B, D and E), and apoptotic cells (cleaved caspase-3 positive) were observed in the samples of all these patients (Fig. 8D and E, Table S4). Quantification of *IL-6* gene expression revealed an up-regulation of this proinflammatory cytokine in the olfactory mucosa of the three patients with high viral load, but not in the case #6, which nevertheless presented SARS-CoV-2 antigens in the neuroepithelium (Table S4). *IL-6* expression in the patients with persistent signs of COVID-19 were similar to those of patients with acute COVID-19 (Tables S1-S2, Fig. 8C). No changes were observed in *Ccl5*, *Cxcl10*, *Isg20* and *Mx1* transcripts (Table S4).

Patient #7, exhibiting long-lasting COVID-19 symptoms but normal sense of smell, had no detectable SARS-CoV-2 RNA in nasopharyngeal samples at inclusion. However, this patient had detectable SARS-CoV-2 RNA in the nasal cytobrush sample using the RT-qPCR SYBR technique and a quantifiable viral genomic RNA load (1.88 10^5^ RNA copies/μL; Taqman technique), but neither subgenomic RNA nor SARS-CoV-2 antigen (NP protein) was detected in the olfactory mucosa by immunostaining (Fig. 8D, Table S4). Having found that a prolonged carriage of SARS-CoV-2 in the olfactory mucosa is not necessarily associated with loss of smell, we investigated immune responses in the olfactory neuroepithelium of this patient. Although *IL-6* expression was high in this patient’s sample, very few myeloid cells (Iba1+) were observed and no cell death was detected in harvested cells (Fig. 8E, Table S4).

Altogether, these data indicate that the olfactory neuroepithelium from patients with persistent loss of smell remains infected, with continual SARS-CoV-2 RNA in all of them, and persevering inflammation. Because sustained infection was also found in a patient with long-lasting COVID-19 symptoms and normal sense of smell, but with seemingly less severe inflammation of the olfactory mucosa, we hypothesize that persistence of COVID-19 associated loss of smell is linked to the inflammation caused by persistent infection.

## DISCUSSION

By combining investigations of COVID-19-associated olfactory function loss in patients and experimentally-infected hamsters, both naturally permissive to SARS-CoV-2 infection, we demonstrate that multiple cell types of the olfactory neuroepithelium are infected during the acute phase, at the time when loss of smell manifests, and that protracted viral infection and inflammation in the olfactory neuroepithelium may account for prolonged hyposmia/anosmia.

Olfactory mucosa cytological sampling collected from acute or chronically patients with COVID-19 with olfactory function loss revealed the presence of the SARS-CoV-2 in 100% of patients (*n*=11**)** whereas the virus was undetected by RT-qPCR performed at inclusion on conventional nasopharyngeal swabs. Therefore, diagnosing SARS-CoV-2 infection in olfactory mucosa sampled by use of nasal cytobrushes is a more sensitive approach, at least in patients with olfactory function loss, than conventional nasopharyngeal samples. This presence of SARS-CoV-2 RNA and proteins may influence care management of patients with COVID-19 as it may play a role in virus transmission from patients who are thought to be viral-free based on conventional testing, particularly in individuals with mild or no symptoms.

We therefore confirm that SARS-CoV-2 has a tropism for the olfactory mucosa (*33*) and, most importantly, we demonstrate that it can persist locally, not only a few weeks after general symptoms resolution (*34*–*36*), but several months in OSNs. Hence, we found that SARS-CoV-2 persists in the olfactory mucosa of patients with prolonged olfactory function loss, up to 6 months after initial diagnosis. Sampling of the olfactory mucosa revealed viral RNA as well as viral antigens, indicating that long-lasting olfactory function loss in these patients correlates with persistence of both viral infection and inflammation, as shown by high expression of inflammatory cytokines including IL-6, and the presence of myeloid cells in cytological samples. Although reinfection by SARS-CoV-2 could not be formally excluded in these patients (*31*), the fact that they showed uninterrupted olfactory dysfunction since the onset of the disease, as well as the very low incidence of COVID-19 in France at the time of inclusion, does not support this option. However, there is no absolute correlation between long-term virus carriage and clinical signs, as we also reported here one long COVID-19 case presenting with persistent viral infection without concomitant loss of smell (but with concomitant dysgeusia). The most likely explanation to this observation is the variability of inflammation associated to long-term SARS-CoV-2 carriage, which can be fully asymptomatic (*37*, *38*) or associated with local inflammation and symptoms, such as in the patients who participated in this study. In addition, in the single patient with COVID-19 but without loss of smell, no cell death or immune cells were observed in the olfactory mucosa, but IL-6 was elevated. Therefore, it will be important to formally evaluate the extent of acute inflammation, immune cell infiltration, and cell and tissue damages among larger cohorts of patients with varying degrees of smell loss to extend our observations made on human olfactory mucosa, and to identify the most important determinants of anosmia.

To further study anosmia and the inflammatory process in the olfactory system in the context of COVID-19, we used the golden hamster as an animal model for COVID-19. We show that intranasal SARS-CoV-2 inoculation in hamsters leads to infection of OSNs and induces anosmia, accurately recapitulating what is observed in patients, both clinically and histopathologically. Infection of OSNs in SARS-CoV-2-inoculated hamsters has been reported in experiments using similar viral inoculum (*26*), but not when the inoculum was lower (*21*), suggesting a dose-dependent susceptibility of OSNs to infection (*5*, *27*, *39*, *40*). Olfactory neurons express Neuropilin-1, a membrane protein involved in SARS-CoV-2 cell entry (*5*), which may account for olfactory neuron infection. However, they almost do not express SARS-CoV-2 primary receptor ACE2 (*41*), as opposed to sustentacular cells. Infection of OSNs may happen after infection of adjacent sustentacular cells and horizontally spread of the virus to a neighboring cell. Furthermore, the infiltration of immune cells and the disruption of tissue architecture may contribute to the dissemination of the virus across cells in the olfactory epithelium.

As observed in the tracheal epithelium (*42*), infection of the neuroepithelium is associated with cilia loss of the OSNs. Once cilia are restored in the late phase of infection (14 dpi in hamsters), olfaction resumes. Given that odorant receptors accumulate on sensory cilia in the olfactory epithelium, loss of smell in COVID-19 may be explained by the viral‐induced cell death of olfactory neurons, or/and also by the disruption of their ciliary structure, preventing the detection of odorant molecules. Although not mutually exclusive, the latter hypothesis would explain why during COVID-19 the onset of anosmia is very sudden, prior to any other respiratory symptoms. Overall, anosmia likely reflects an infection-associated sensorineural dysfunction, that might involve a substantial inflammatory process, neuronal infection, deciliation and cell death, rather than a simple nostril obstruction or tissue edema. The molecular mechanisms underlying neuronal dysfunction remain to be deciphered, but we recently reported that ivermectin strongly reduces loss of smell and is associated to a decrease in inflammation in the nasal turbinates of infected hamsters, without decreasing viral load (*43*). Therefore, immune responses induced by olfactory neurons infection might play a role in the onset and persistence of anosmia, and could explain why some infected individuals with SARS-CoV-2 in their nasal cavity never develop anosmia.

We also found that this inflammatory process that takes place in the nasal cavity spreads to the olfactory bulb. This inflammatory transcriptional signature, as shown by RNA Seq and confirmed by qPCR for *Il-6***,** type I IFN (*Ifn-β*) and *Cxcl10* is consistent with the recent neuropathological description of deceased patients with COVID-19, where microgliosis was seen in the olfactory bulb (*12*). Importantly, the fact that similar neuropathological alterations are observed in patients with COVID-19 and infected hamsters implies that SARS-CoV-2 infection is likely the cause rather than a consequence of intensive care provided to patients with COVID-19, as previously hypothesized (*44*).

Although several viruses are known to invade and disseminate into the brain, whether SARS-CoV-2 does so is highly debated. For instance, viral RNA has been detected in the cerebrospinal fluid and other brain tissues collected from patients who died from COVID-19 (*12*), but the neuropathological relevance of these observations remains unclear (*6*, *13*, *45*). The potential SARS-CoV-2 portals of entry to the CNS are (*i*) retrograde neuroinvasion (via olfactory sensory neurons, glossopharyngeal and/or vagal nerve), (*ii*) via the blood-brain barrier endothelium (*13*, *46*) and (*iii*) via peripheral immune cells infiltration (such as T-cells or peripheral macrophages). Although this does not rule out other routes, our study indicates that SARS-CoV-2 indeed does invade the CNS via the retrograde olfactory pathway. Importantly, in addition to the olfactory bulb, viable SARS-CoV-2 was also detected in more remote brain areas of infected hamsters, such as the cerebral cortex and the brainstem, yet without clear visualization of viral antigens. Similarly, viral RNA or protein were observed in the brainstem of patients with COVID-19 (*12*, *39*), the location of central cardiorespiratory nuclei. Infection of these areas might contribute to the pathogenesis of the respiratory distress reported in patients with COVID-19 and this study therefore constitutes an important step toward elucidating COVID-19-associated putative neurological dysfunctions. Whether neuronal structures are directly targeted by SARS-CoV-2, as opposed to damage by systemic immune responses, is of particular clinical relevance since these two scenarios would require different therapeutic strategies.

Limitations of our study includes the small sample size of the human cohort, especially for patients with COVID-19 without loss of smell. Extending these investigations to a larger group of patients, with varying degree of loss of smell, would allow to precise these first findings. Moreover, this study would benefit from additional approaches to determine the potential dysfunction of brain structures such as the olfactory bulb, by using metabolic PET scan imaging as well as in-depth neurosensory and neurocognitive assessment. Finally, the discovered persistence of SARS-CoV-2 in the olfactory mucosa of patients with long-lasting COVID-19 symptoms requires future validation in larger groups of people from different countries and to set-up new animal models of long COVID.

The persistence of long-lasting COVID-19 symptoms is an important and topical issue as the pandemic continues (*47*). This study demonstrates a persistent loss of olfactory function in humans with SARS-CoV-2, for multiple months, lasting as long as the virus remains in the same microenvironment. This might result from direct damage to the OSNs which detect odor in the olfactory epithelium. Further, it provides evidence of SARS-CoV-2 retrograde neuroinvasion via the olfactory route leading to neuroinflammation, and shows the association between viral presence in the olfactory epithelium and anosmia, in both acute (hamsters and humans) and long-lasting in COVID-19 patients. The findings we obtained are clinically relevant in the care to patients with COVID-19, since olfactory function loss could be regarded as a sensitive sign of persistent viral infection, and should be considered in patient management.

## MATERIALS AND METHODS

### Study design

The main objective of this study was to investigate SARS-CoV-2 neuroinvasion in humans and the hamster model and to decipher the molecular mechanisms involved. Human olfactory mucosa samples were obtained from a total of 16 living individuals (12 patients with COVID-19, 4 healthy controls) at the ENT department of Lariboisière Hospital, Paris, France, from May to October 2020. All these individuals were recruited in the CovidSmell study (Study of the Pathogenesis of Olfactory Disorders in COVID-19, ClinicalTrials.gov identifier NCT 04366934). This prospective, case-control study is a non-trial, non-drug study, qualified as exploratory, descriptive, monocentric, in an adult population. This study received the approval from the ethical committee “Comité de Protection des Personnes SUD EST IV” under reference 20.04.15.64936 and is compliant with French data protection regulations. All animal experiments were performed according to the French legislation and in compliance with the European Communities Council Directives (2010/63/UE, French Law 2013–118, February 6, 2013). The Animal Experimentation Ethics Committee (CETEA 89) of the Institut Pasteur approved this study (200023; APAFIS#25326-2020050617114340 v2.) before experiments were initiated. All animals were randomized to the different experimental groups. Sample sizes were chosen empirically to ensure adequate statistical power. Investigators were not blinded with respect to the origin of the samples. For analysis of the human mucosa, two nasal samples were collected for each participant (one sample per nostril; one immediately frozen and the other one fixed in formalin). Each nasal sample was analyzed three times for SARS-CoV-2 assay by PCR (frozen samples) and immunohistochemistry (formol samples). Nasal samples of two patients and two controls were also titrated for SARS-CoV-2 on cell cultures. In animal experiments, at least four males were used for each time point and replication was performed with the same number of females. Animal samples were analyzed three times for SARS-CoV-2 assay by PCR (frozen samples), immunohistochemistry (formol samples) and titrated by cell cultures (frozen samples). Olfactory mucosa samples of two controls and five SARS-CoV-2 -infected hamsters were analyzed for SARS-CoV-2 by scanning electron microscopy, and 2 control and 3 infected hamsters for immunofluorescence. For microscopy quantification, the value for each animal is the average of 4-8 independently quantified fields. All measurements in humans and hamsters were included in our analysis. Primary data are provided in the figures or the Supplementary Materials.

### Participants

Subjects with recent olfactory function loss consulting in the Lariboisière hospital (Paris, France) in the context of the COVID-19 screening care for a suspected or confirmed SARS-CoV-2 infection were included during spring and summer 2020. We also recruited subjects with long-lasting/recurrent loss of smell after onset of acute COVID-19 symptoms. Those patients were recruited at the Hotel Dieu Hospital clinic dedicated for patients with long-COVID-19. Among these patients, we included a control patient without loss of smell, consulting for long-term dysgeusia, vertigo, paresthesia, evocative of COVID-19-associated neuroinvasion. Acute illness for those patients was always symptomatic and was either certain if virologically documented (at least one positive SARS-CoV-2 RT-PCR result or positive SARS-CoV-2 serology with IgG and/or IgM antibodies), or probable if at least one major criteria: anosmia/ageusia, contact with a PCR conformed case, typical bilateral pneumonia on CT scan, or three minor criteria among: fever, headaches, fatigue, myalgias, cough, chest pain, chest tightness, unexplained tachycardia, diarrhea, chilblains. We also recruited in this study control subjects consulting in the Ear, Nose and Throat department at the Lariboisière hospital (Paris, France) with no biologically confirmed COVID-19 or suspected COVID-19 in the past 8 weeks, and no symptoms suggestive of COVID-19 or another respiratory disease and therefore no recent taste and smell function loss.

All the research participants were included at the Lariboisière Hospital, Paris. They received an oral and written information about the research. Informed consent was obtained by the investigator before any intervention related to the research. The Covidsmell study was performed according to the approved protocol. All patients had a detailed standardized clinical and rhinological examination performed by a certified ENT physician. Following measures were performed at inclusion: *i*) Taste and olfactory function evaluation by a self-questionnaire taste and smell survey (TTS) (*48*), and a visual analog scale (VAS) (*49*), and *ii*) Nasal brushing for collection of neuroepithelium cells and olfactory mucus. The participants self-assessed their smell and taste perception using a 100-mm VAS, where 0 mm indicated the inability to smell or taste and 100 mm indicated normal smell or taste perception (*49*).

### Human nasal cytobrushes sampling

A certified ENT physician sampled olfactory mucosa of each participant by nasal brushing with safety precautions and after local xylocaine application (Lidocaine 5%) following the method previously described (*30*). Briefly, sampling was performed with a sterile 3.5 mm endocervical brush (02.104, Gyneas, Goussainville, France) inserted and gently rolled five times around the inside of both nostrils (360°). Swabs (one per nostril) were placed on ice immediately following collection, and frozen at -80°C or put in formalin solution 10% neutral buffered (HT-5011-1CS, Sigma).

### Production and titration of SARS-CoV-2 virus

The strain 2019-nCoV/IDF0372/2020 (EVAg collection, Ref-SKU: 014V-03890) was provided by Sylvie Van der Werf, Institut Pasteur, Paris. Viral stocks were produced on Vero-E6 cells infected at a multiplicity of infection of 1.10^−4^ PFU (plaque-forming units). The virus was harvested 3 days post-infection, clarified and then aliquoted before storage at -80°C. Viral stocks were titrated on Vero-E6 cells by classical plaque assay using semisolid overlays (Avicel, RC581-NFDR080I, DuPont) (*50*).

### SARS-CoV-2 model in hamsters

Male and female Syrian hamsters (*Mesocricetus auratus*) of 5-6 weeks of age (average weight 60-80 g) were purchased from Janvier Laboratories and handled under specific pathogen-free conditions. Hamsters were housed by groups of 4 animals in isolators in a Biosafety level-3 facility, with *ad libitum* access to water and food. Before any manipulation, animals underwent an acclimation period of one week. Animal infection was performed as previously described with few modifications (*23*). Briefly, the animals were anesthetized with an intraperitoneal injection of 200 mg/kg ketamine (Imalgène 1000, Merial) and 10 mg/kg xylazine (Rompun, Bayer), and 100 μL of physiological solution containing 6.10^4^ PFU (plaque-forming units) of SARS-CoV-2 (strain 2019-nCoV/IDF0372/2020, from Pr Sylvie van der Werf) was administered intranasally to each animal (50 μL/nostril). Mock-infected animals received the physiological solution only. Infected and mock-infected animals were housed in separated isolators and all hamsters were followed-up daily when the body weight and the clinical score were noted. The clinical score was based on a cumulative 0-4 scale: ruffled fur, slow movements, apathy, stress when manipulated. At predefined time-points post-infection, animals were submitted to behavioral tests or euthanized, when samples of nasal turbinates, trachea, lungs and the brain (separated in olfactory bulbs, cerebellum, cortex and brainstem) were collected, immediately frozen at -80°C or formalin-fixed after transcardial perfusion with a physiological solution containing 5.10^3^ U/mL heparin (choay, Sanofi) followed by 4% neutral buffered formaldehyde.

### Behavioral tests

All behavioral assessment was performed in isolators in a Biosafety level-3 facility that we specially equipped for that.

*Sucrose preference test.* We measured taste in hamsters by a sucrose preference test based on a two-bottle choice paradigm which paired 2% sucrose with regular water (*51*). A reduction in the sucrose preference ratio in experimental infected relative to mock animal is indicative of taste abnormalities. After 6 hours water deprivation, we conducted an individual overnight testing which corresponds to a natural activity period of the hamster. The preference was calculated using the following formula: preference = sucrose intake/total intake × 100%. The total intake value is the sum of the sucrose intake value and the regular water intake

*Buried food finding test.* To assess olfaction, we used the buried food finding test as previously described (*52*) with few modifications. Hamsters were used only once for each test. Four days before testing, Hamsters received chocolate cereals (Coco pops, Kellogg’s) that they ate within one hour. Twenty hours before testing, hamsters were fasted and then individually placed into a fresh cage (37 × 29 × 18 cm) with clean standard bedding for 20 min. Hamsters were placed in another similar cage for 2 min when about 10-12 pieces of cereals were hidden in 1.5 cm bedding in a corner of the test cage. The tested hamster was then placed in the opposite corner and the latency to find the food (defined as the time to locate cereals and start digging) was recorded using a chronometer. The test was carried out during a 15 min period. As soon as food was uncovered, hamsters were removed from the cage. One minute later, hamsters performed the same test but with visible chocolate cereals, positioned upon the bedding.

### Scanning electron microscopy

For scanning electron microscopy, following animal transcardial perfusion in PBS then 4% neutral buffered formaldehyde, hamster whole heads and lungs where fixed in formalin solution 10% neutral buffered (HT-5011-1CS, Sigma), for one week at 4°C to allow neutralization of the virus. Lung and olfactory epithelium small samples were then finely dissected and post-fixed by incubation in 2.5% glutaraldehyde in 0.1 M cacodylate buffer for 1 hour at room temperature then 12 hours at 4°C. The samples were washed in 0.1 M cacodylate then several times in water and processed by alternating incubations in 1% osmium tetroxide and 0.1 M thiocarbohydrazide (OTOTO method), as previously described (*53*). After dehydration by incubation in increasing concentrations of ethanol, samples were critical point dried, mounted on a stub, and analyzed by field emission scanning electron microscopy with a Jeol JSM6700F operating at 3 kV.

### Immunofluorescence

Tissues from PFA-perfused animals were post-fixed one week in PFA 4%, and olfactory brushes from patients were kept in PFA until further use. After post-fixation, hamster whole heads (without skin and lower jaw) were decalcified in TBD-2 (6764003, ThermoFisher) for 3-5 days, then sagitally cut in half and rinsed in PBS. Organs or brushes were then washed in PBS and dehydrated in 30% sucrose. They were then embedded in O.C.T compound (4583, Tissue-Tek), frozen on dry ice and cryostat-sectioned into 20 μm-thick (hamster organs) or 14 μm-thick (brushes) sections. Sections were rinsed in PBS, and epitope retrieval was performed by incubating sections for 20min in citrate buffer pH 6.0 (C-9999, Sigma-Aldrich) at 96°C for 20min, or overnight at 60°C for whole head sections as they are prone to detaching from the slides. Sections were then blocked in PBS supplemented with 10% goat serum, 4% fetal calf serum and 0.4% Triton X-100 for 2h at room temperature, followed by overnight incubation at 4°C with primary antibodies: rat anti-CD11b (1/100, 550282, BD-Biosciences), rabbit anti-SARS-CoV nucleoprotein (1/500, provided by Dr Nicolas Escriou, Institut Pasteur, Paris), mouse anti-OMP (1/250, sc-365818, Santa-Cruz), chicken anti-Iba1 (1/500, 234006, SynapticSystems), mouse anti-Tuj1 (1/250, MA1-118, ThermoFisher**),** mouse anti PGP9.5 (1/500, ab8189, Abcam), rabbit anti-cleaved caspase 3 (1/250, Cell Signaling, Asp 175). After rinsing, slides were incubated with the appropriate secondary antibodies (1/500: goat anti-rat Alexa Fluor 546, A11081; goat anti-rabbit Alexa Fluor 488, A11034; goat anti-mouse IgG2a Alexa Fluor 546, A21133; goat anti-chicken Alexa Fluor 647, A32933, Invitrogen) for 2 hours at room temperature. All sections were then counterstained with Hoechst (H3570, Invitrogen), rinsed thoroughly in PBS and mounted in Fluoroumont-G (15586276, Invitrogen) before observation with a Zeiss LM 710 inverted confocal microscope. Quantification of cells was performed using ImageJ in a semi-automated manner.

### RNA isolation and transcriptional analyses by quantitative PCR from Human nasal cytobrushes

Frozen cytobrushes samples were incubated with Trizol (15596026, Invitrogen) during 5 min and the total RNA was extracted using the Direct-zol RNAMicroPrep Kit (R2062, Zymo Research). The presence of the SARS-CoV-2 in these samples was evaluated by one-step qRT-PCR in a final volume of 25 μL per reaction in 96-well PCR plates using a thermocycler (7500t Real-time PCR system, Applied Biosystems, Applied Biosystems). Briefly, 5 μL of diluted RNA (1:10) was added to 20μL of Superscript III Platinum One-Step qRT-PCR mix (Invitrogen 11746-100) containing 12.5 μL reaction mix, 0.4 μL 50 mM MgSO_4_, 1.0 μL superscript RT and 6.1 μL of nuclease-free water containing the nCoV_IP2 primers targeting the RdRp gene (nCoV_IP2-12669Fw: 5′-ATGAGCTTAGTCCTGTTG-3′; nCoV_IP2-12759Rv: 5′-CTCCCTTTGTTGTGTTGT-3′) at a final concentration of 1 μM (*54*). The amplification conditions were as follows: 1 cycle of 55°C for 20 min, 1 cycle of 95°C for 3 min, 50 cycles of 95°C for 15 s and 58°C for 30 s, 1 cycle of 40°C for 30 s; followed by a melt curve, from 60 °C to 95 °C. The viral load quantification in these samples was assessed using a Taqman one-step qRT-PCR (Invitrogen 11732-020), with the same nCoV_IP2 primers and the nCoV_IP2 probe (5′-FAM-AGATGTCTTGTGCTGCCGGTA-3′-TAMRA) at a final concentration of 1 μM (*54*).

The detection of genomic and subgenomic SARS-CoV-2 RNA was based on the E gene (*54*) using a Taqman one-step qRT-PCR (Invitrogen 11732-020): to detect the genomic RNA we used the E_sarbeco primers and probe (E_Sarbeco_F1 5′-ACAGGTACGTTAATAGTTAATAGCGT-3′; E_Sarbeco_R2 5′-ATATTGCAGCAGTACGCACACA-3′; E_Sarbeco_Probe FAM-5′-ACACTAGCCATCCTTACTGCGCTTCG-3′-TAMRA). The detection of subgenomic SARS-CoV-2 RNA was achieved by replacing the E_Sarbeco_F1 primer by the CoV2sgLead primer (CoV2sgLead-Fw 5′-CGATCTCTTGTAGATCTGTTCTC-3′). A synthetic gene encoding the PCR target sequences was ordered from Thermo Fisher Scientific. A PCR product was amplified using Phusion High-Fidelity DNA Polymerase (Thermo Fisher Scientific) and in vitro transcribed by means of Ribomax T7 kit (Promega). RNA was quantified using Qubit RNA HS Assay kit (Thermo Fisher scientific), normalized and used as a positive control to quantify RNA absolute copy number.

Total RNA from human cytobrushes was also reverse transcribed to first strand cDNA using the SuperScript IV VILO Master Mix (11766050, Invitrogen). To quantify host inflammatory mediators’ transcripts (IL-6, CXCL10, CCL5, Mx1 and ISG20), qPCR was performed in a final volume of 10 μL per reaction in 384-well PCR plates using a thermocycler (QuantStudio 6 Flex, Applied Biosystems). Briefly, 2.5 μL of cDNA (12.5 ng) was added to 7.5 μL of a master mix containing 5 μL of Power SYBR green mix (4367659, Applied Biosystems) and 2.5 μL of nuclease-free water containing predesigned primers (#249900, Qiagen; QuantiTect Primer Assays *IL-6*: QT00083720; *CXCL10*: QT01003065; CCL5: QT00090083; *Mx1*: QT00090895; *ISG20*: QT00225372**;** *OMP*: QT00237055**;** and *GAPDH*: QT00079247). The amplification conditions were as follows: 95°C for 10 min, 45 cycles of 95°C for 15 s and 60°C for 1 min; followed by a melt curve, from 60 °C to 95 °C. Variations in the gene expression were calculated as the n-fold change in expression in the tissues compared with the tissues of the control #1.

### RNA isolation and transcriptional analyses by quantitative PCR from Golden hamsters’ tissues

Frozen tissues were homogenized with Trizol (15596026, Invitrogen) in Lysing Matrix D 2 mL tubes (116913100, MP Biomedicals) using the FastPrep-24 system (MP Biomedicals**)** and the following scheme: homogenization at 6.5 m/s during 60 s, and centrifugation at 12.000 × g during 2 min at 4°C. The supernatants were collected and the total RNA was then extracted using the Direct-zol RNA MicroPrep Kit (R2062, Zymo Research: olfactory bulb, trachea and nasal turbinates) or MiniPrep Kit (R2052, Zymo Research: lung, brainstem, cerebral cortex and cerebellum) and reverse transcribed to first strand cDNA using the using the SuperScript IV VILO Master Mix (11766050, Invitrogen). qPCR was performed in a final volume of 10 μL per reaction in 384-well PCR plates using a thermocycler (QuantStudio 6 Flex, Applied Biosystems). Briefly, 2.5 μL of cDNA (12.5 ng) was added to 7.5 μL of a master mix containing 5 μL of Power SYBR green mix (4367659, Applied Biosystems) and 2.5 μL of nuclease-free water with the nCoV_IP2 primers (nCoV_IP2-12669Fw: 5′-ATGAGCTTAGTCCTGTTG-3′; nCoV_IP2-12759Rv: 5′-CTCCCTTTGTTGTGTTGT-3′) at a final concentration of 1 μM. The amplification conditions were as follows: 95°C for 10 min, 45 cycles of 95°C for 15 s and 60°C for 1 min; followed by a melt curve, from 60 °C to 95 °C. Viral load quantification in hamster tissues was assessed by linear regression using a standard curve of eight known quantities of plasmids containing the *RdRp* sequence (ranging from 10^7^ to 10° copies). The threshold of detection was established as 200 viral copies/μg of RNA. The Golden hamsters’ gene targets were selected for quantifying host inflammatory mediators’ transcripts in the tissues using the *Hprt* (hypoxanthine phosphoribosyltransferase) and the *γ-actin* genes as reference (Table S6). Variations in the gene expression were calculated as the n-fold change in expression in the tissues from the infected hamsters compared with the tissues of the uninfected ones using the 2^-ΔΔCt^ method (*55*).

### Viral titration in Human nasal cytobrushes and in Golden hamsters’ brains

Frozen cytobrushes samples of the patients #14 and #15 and the controls #3 and #4 were incubated with 1 mL of ice-cold DMEM supplemented with 1% penicillin/streptomycin (15140148, Thermo Fisher) during 5 min. Frozen fragments of hamster tissues (lung, olfactory bulb, brainstem, cerebral cortex, cerebellum) were weighted and homogenized with 1 mL of ice-cold DMEM supplemented with 1% penicillin/streptomycin in Lysing Matrix M 2 mL tubes (116923050-CF, MP Biomedicals) using the FastPrep-24 system (MP Biomedicals) and the following scheme: homogenization at 4.0 m/s during 20 s, incubation at 4°C during 2 min, and new homogenization at 4.0 m/s during 20 s. The tubes were centrifuged at 10.000 × g during 1 min at 4°C. The supernatants were titrated on Vero-E6 cells by classical plaque assays using semisolid overlays (Avicel, RC581-NFDR080I, DuPont) (*50*). RNA was isolated from the supernatants using Trizol LS (10296028, Invitrogen) and the Direct-zol RNA MicroPrep Kit (R2062, Zymo Research) as described above.

### Transcriptomics analysis in Golden hamsters’ olfactory bulb

RNA preparation was used to construct strand specific single end cDNA libraries according to manufacturers’ instructions (Truseq Stranded mRNA sample prep kit, Illumina). Illumina NextSeq 500 sequencer was used to sequence libraries. The complete RNA-seq analysis approach is described in the Supplemental information.

### Statistical Analysis

Statistical analysis was performed using Stata 16 (StataCorp LLC, Texas, USA) and Prism software (GraphPad, version 8, San Diego, USA), with *p* < 0.05 considered significant. Quantitative data were compared across groups using Mann-Whitney non-parametric test. Categorical data was compared between groups using Fisher exact test. Associations between the viral load, the olfactory and taste scores, the expression of cytokines, and the time from the first disease symptom were estimated with Spearman non-parametric test. In the animal experiences, time to event were analyzed using Kaplan-Meier estimates and compared across groups using the Logrank test. The degree of markers expression at different dpi were compared to the expression pre-infection using Kruskal-Wallis followed by the Dunn’s multiple comparison test for unmatched data.

## Supplementary Materials

stm.sciencemag.org/cgi/content/full/scitranslmed.abf8396/DC1 Materials and Methods. Fig. S1. General symptoms and infected cell types in olfactory mucosa of patients with COVID-19 at the acute phase. Fig. S2. Experimental design of the experiments and complementary behavioral tests with golden hamsters. Fig. S3. SARS-CoV-2 induces loss of ciliation in the golden hamster olfactory epithelium Fig. S4. Cell types infected by SARS-CoV-2 in hamster olfactory mucosa and subsequent cell death. Fig. S5. Clinical and virologic profiles from patients with persistent olfactory dysfunction post-COVID-19 compared to patients with COVID-19 exhibiting loss of smell at early onset and controls Table S1. Features at inclusion of the participants with recent loss of smell associated to COVID-19. Table S2. SARS-CoV-2 titer in the olfactory mucosa in two patients with recent loss of smell associated to COVID-19. Table S3. Characteristics of smell and taste abnormalities at inclusion of the participants with recent loss of smell associated to COVID-19. Table S4. Individual features at inclusion of the participants with persistent olfactory dysfunction. Table S5. Characteristics of smell and taste abnormalities at inclusion of the participants with persistent olfactory dysfunction. Table S6. Primer sequences used for qPCR in the golden hamster tissues. Data File S1. Raw data except RNA-seq (provided as supplementary Excel file). Data File S2. Raw data for RNA-seq/Fig. 7 (provided as supplementary Excel file).
